# Unresolved Complete Heart Block in a Patient Treated for Euthyroid Graves’ Disease: A Diagnostic Dilemma

**DOI:** 10.7759/cureus.75746

**Published:** 2024-12-15

**Authors:** Tharun Adithya Natesan, Mehereen Murshed, Sandhya Abraham, Cornelius Fernandez

**Affiliations:** 1 Internal Medicine, Nottingham University Hospital NHS Trust, Nottingham, GBR; 2 Internal Medicine, Queen Elizabeth Hospital Birmingham, Birmingham, GBR; 3 Haematology, Addenbrooke's Hospital, Cambridge University Hospitals NHS Foundation Trust, Cambridge, GBR; 4 Diabetes and Endocrinology, United Lincolnshire Hospital NHS Trust, Lincoln, GBR

**Keywords:** bradyarrhythmias, complete heart block, graves' disease, hyperthyroid, permanent pacemaker (ppm)

## Abstract

The cardiovascular implications of thyroid disease have been recognized as one of the most characteristic signs that result from the effect of thyroid hormone (TH). Both hyperthyroidism and hypothyroidism produce changes in cardiac contractility, myocardial oxygen consumption, cardiac output, blood pressure, and systemic vascular resistance.

The bradyarrhythmias, including atrioventricular block and sick sinus syndrome, are exceedingly rare in hyperthyroidism.Studies have stated that atrioventricular block complicating a hyperthyroid state is the rarest and most under-studied cardiac complication. Only a few case reports have described this association, which is commonly seen with acute inflammatory states, infections, or medications. Here, we present the case of a 16-year-old girl who presented with thyrotoxicosis and was incidentally found to have asymptomatic complete heart block (CHB) in the setting of newly diagnosed Graves’ disease. This case report explores the course of her treatment and possible causes given the rarity of presentation.

## Introduction

Thyroid hormones (THs) are said to modulate every component of the cardiovascular system, essential for cardiovascular development and function. As such, cardiac complications due to TH abnormalities are common [1].

The hypothalamic-pituitary-thyroid axis is one of several hormone-regulatory systems from the hypothalamus to the pituitary, culminating in the peripheral target organs. The secretion of hormones by the anterior lobe of the pituitary, which consists of at least five different types of cells, each secreting a different hormone, is under the regulation of the hypothalamus. The paraventricular nucleus of the hypothalamus produces thyrotropin-releasing hormone (TRH); this stimulates the secretion of pituitary thyrotropin (thyroid-stimulating hormone (TSH)) from the thyrotropic cells in the anterior pituitary gland. Thyroid-stimulating hormone, in turn, stimulates thyroid follicular cells to release triiodothyronine (T3) and thyroxine (T4). Normal thyroid function is reliant on a negative feedback control system. The concentration of T3 and T4 in peripheral organs affects the biosynthesis and secretion of TRH in the hypothalamus and TSH in the pituitary [2]. It is well known that TH exhibits a variety of effects on the heart; they raise the heart rate and cardiac contractility, improve the systolic and diastolic function of the heart, and decrease systemic vascular resistance in a resting state [3]. The haemodynamic effects caused by thyroid hormones, whether their factors influence venous return or arteriolar dilatation, reducing peripheral vascular resistance and thus increasing cardiac output, are all critical in cardiac function [4].

The most important cardiac dysfunction associated with patients with hypothyroidism is diastolic dysfunction, exacerbating the symptoms and worsening the prognosis of potential heart failure [5]. It is accepted that typically hypothyroid electrocardiogram (ECG) changes include bradycardia, long PQ segments, low voltage of the QRS complex, flattening, or T-wave inversions. In contrast, arrhythmias, including sinus tachycardia, atrial fibrillation, and shortened PR and QT intervals, tend to be observed in patients in a hyperthyroid state; the most common rhythm disturbance is sinus tachycardia [6]. In rare cases, atrioventricular blockage might be observed in patients with Graves' disease [7].

Patients with hyperthyroidism have enhanced triggered activity and automaticity of the atria and pulmonary veins. Patients with both overt and subclinical hyperthyroidism are associated with an increased risk for tachyarrhythmias, and both groups have nearly a 20% rise in mortality compared to the general population. Though patients with Graves’ disease, as well as toxic nodular goitre, have an increased risk for tachyarrhythmias and congestive cardiac failure, the risk is greater for patients with toxic nodular goitre compared to Graves’ disease, due to the older age group. Moreover, patients with toxic nodular goitre have higher risks of ventricular tachyarrhythmias compared to Graves’ disease patients. On the other hand, pulmonary artery hypertension and cardiomyopathy are common in patients with Graves’ disease. Bradyarrhythmias seen in patients with hyperthyroidism are mostly due to structural heart diseases such as ischaemic cardiomyopathy, inferior myocardial infarction, drugs, infections, and electrolyte disturbances [8].

## Case presentation

A 16-year-old Caucasian girl, with a recent tonsillectomy and a background of lactose and wheat intolerance, was referred to the endocrine clinic for a new diagnosis of thyrotoxicosis. She presented with heat intolerance, sweaty palms, and shaky hands but denied a history of palpitations, diarrhoea, changes in appetite, tiredness, or any weight loss. The young lady was not known to be on any regular medications and had no positive family history of autoimmune illness, hypothyroidism, or hyperthyroidism. 

**Figure 1:**
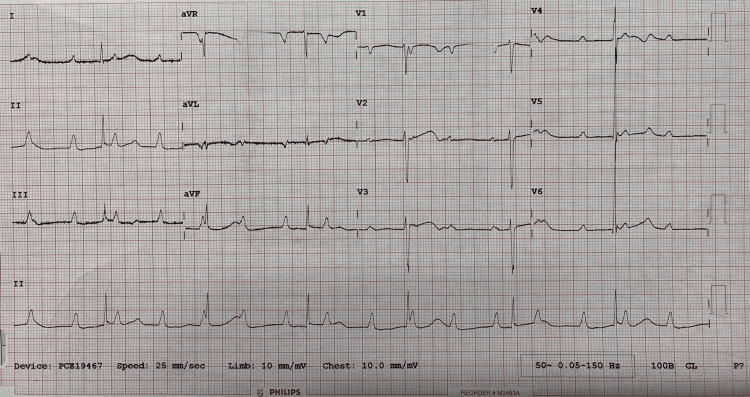
Electrocardiogram depicting complete heart block

On examination, fine tremors and excessive sweating were noted. There were no signs of goitre, thyroid eye changes, or proximal myopathy. Blood tests revealed TSH of <0.01 mU/L, free T4 of 67.4 pmol/L, and free T3 of 20.78 pmol/L (Table 1). Heart rate was 40 beats/minute and regular in rhythm. As bradycardia was an unexpected finding with thyrotoxicosis, an electrocardiogram was done, which showed a complete heart block (CHB) with narrow QRS complexes and a ventricular rate of 40 beats/minute. She was admitted to the acute cardiac unit for continuous cardiac monitoring. Two-dimensional echocardiography showed a structurally normal heart with a left ventricular ejection fraction of 56% (Figure 1).

**Table 1:** The response of thyroid function to anti-thyroid treatment TSH: thyroid-stimulating hormone; T4: thyroxine; T3: triiodothyronine

Thyroid function	Normal values	Day 0	Day 3	Day 5	1 month	4 months
TSH (mU/ml)	0.27-4.5	<0.01	<0.01	<0.01	<0.01	2.6
Free T4 (pmol/l)	11-23	67.4	56.9	69.9	19.4	11.3
Free T3 (pmol/l)	3.1-6.8	20.78	23.43	-	-	-

Given the abovementioned thyroid function tests, she was started on tab carbimazole 30 mg once daily for four weeks and 10 mg once daily to continue thereafter. Beta (β)-blockers were strictly avoided. 

Further evaluation revealed elevated thyroid peroxidase (TPO) and thyroid receptor antibody (TRAb) levels of 158 IU/ml (0-34) and 2.91 U/l (0-1.74), respectively, confirming the diagnosis of Graves’ disease. Ultrasound of the neck further showed diffuse thyroid enlargement, with multiple small echo-poor lesions. 

**Figure 2:**
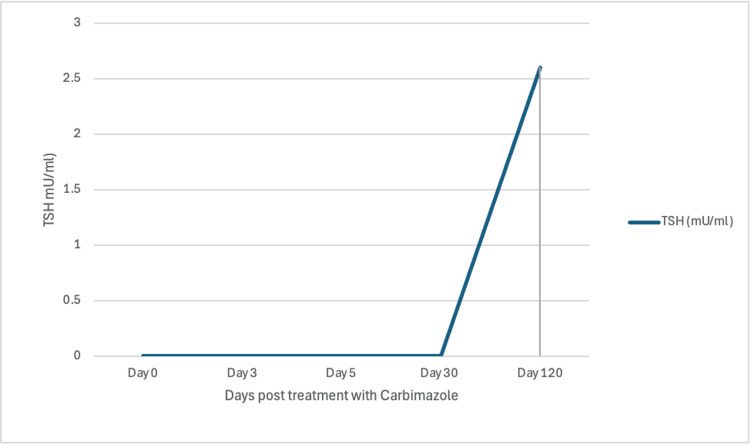
The response of TSH to treatment with carbimazole TSH: thyroid-stimulating hormone

**Figure 3:**
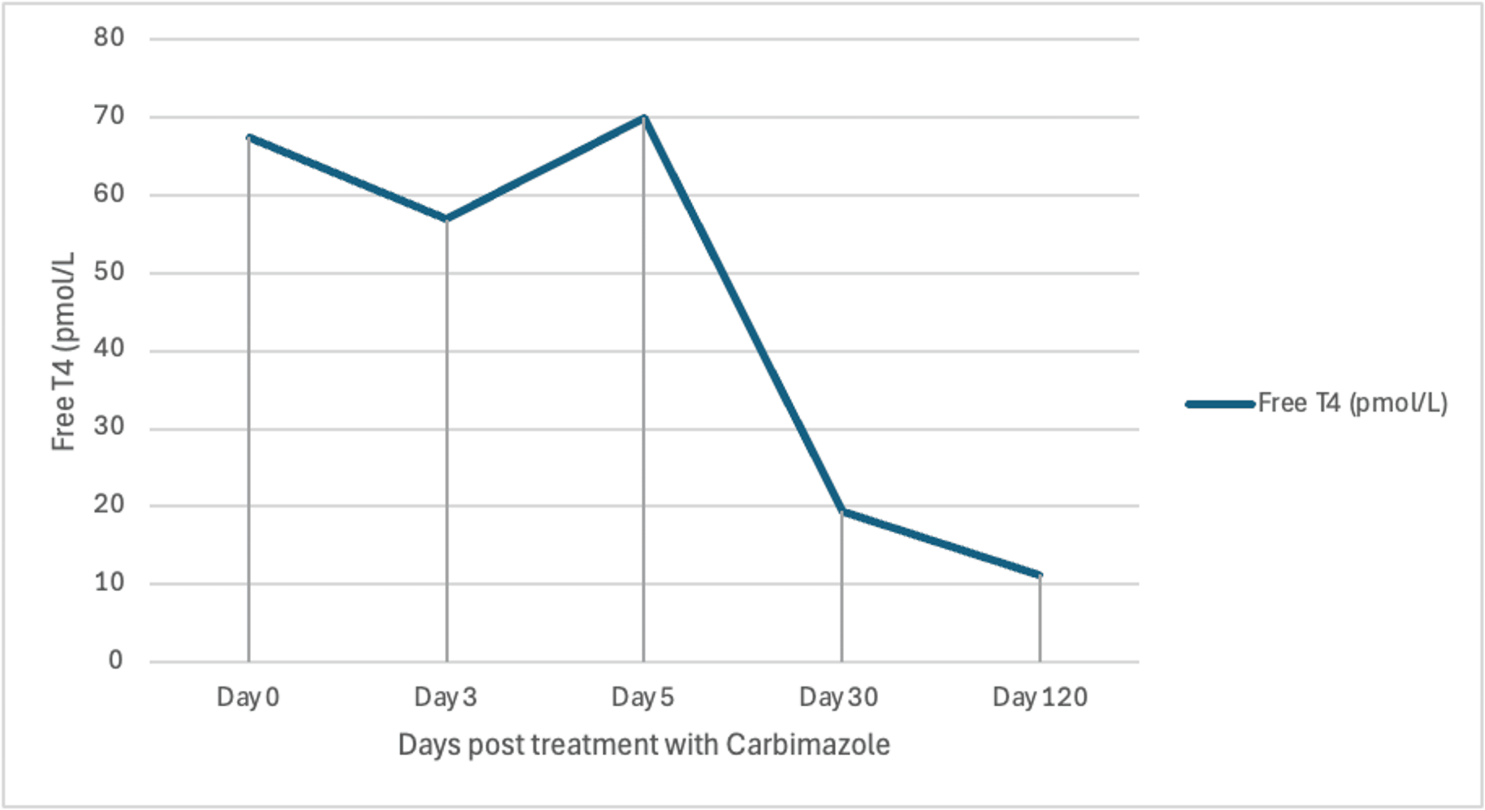
Response of free T4 to treatment with carbimazole T4: thyroxine

The thyroid function tests improved with carbimazole, but her CHB persisted. Table 1 and Figures 2-3 show the response to treatment. During the initial four months of her diagnosis of Graves’ disease with CHB, she remained completely asymptomatic in terms of cardiac symptoms. After a period of observation, she was noted to develop exertional dyspnea. The cardiology team referred her for a permanent pacemaker while they continued to investigate other possible causes for a CHB, including Fabry disease and autoimmune causes. 

## Discussion

Central and peripheral cardiovascular effects of hyperthyroidism

Hyperthyroidism affects the sinoatrial (SA) node, causing tachyarrhythmias, and the cardiac myocytes, causing increased myocardial contractility. Actions of T3 on the heart by altering the production of particular messenger RNA (mRNA) create specific responses that increase cardiac protein synthesis, leading to increased myocardial contractility. Sinus tachycardia is the most common tachyarrhythmia associated with hyperthyroidism, and atrial fibrillation follows soon after. Atrial flutter and supraventricular tachycardia, including paroxysmal atrial tachycardia, are less common, whereas ventricular arrhythmias are extremely rare.

The peripheral effects consist of vasodilation, which results in a fall in peripheral vascular resistance, afterload, and diastolic blood pressure. Vasodilatation reduces renal perfusion, activating the renin-angiotensin-aldosterone system (RAAS), resulting in sodium retention and an increase in circulating volume, preload, and stroke volume. The rise in myocardial contractility along with a fall in peripheral vascular resistance leads to a two- to three-fold rise in cardiac output and systolic hypertension with a wide pulse pressure 8]. However, when the significant rise in circulating volume decreases the myocardial contractile reserve, high-output heart failure develops in 6% of hyperthyroidism patients [9]. A further 1% of hyperthyroidism patients develop thyrotoxic cardiomyopathy mediated by tachyarrhythmias, autoimmune myocarditis, or lymphocytic myocarditis [10]. 

Tachyarrhythmias associated with hyperthyroidism

Atrial Fibrillation

The most prevalent cardiovascular manifestations of hyperthyroidism are sinus tachycardia and atrial fibrillation, as mentioned before. Among patients with hyperthyroidism, patients with toxic nodular goitre exhibit a higher incidence of atrial fibrillation compared to patients with Graves' disease. Autoantibodies towards the beta 1 (β1)-adrenergic and the M2 muscarinic receptors (in Graves’ disease) and elevated TH levels (in Graves’ disease and toxic nodular goitre) increase the risk for atrial fibrillation [11]. The TSH levels ≤ 0.1 mIU/L are associated with a three-fold rise in the risk of atrial fibrillation [12], which in turn is associated with significant cardiovascular morbidity and mortality. 

The overall prevalence of atrial fibrillation in hyperthyroidism patients was 8.3%, with a higher prevalence noted in men than women (12.1% vs. 7.6%). The prevalence was higher (10%-20%) in patients above 60 years, whereas it was lower (1%-5%) in patients between 40 and 60 years, and lowest (<1%) in patients under 40 [13]. Other factors associated with a high risk of atrial fibrillation in hyperthyroidism include the presence of ischaemic heart disease, congestive cardiac failure, and valvular heart disease [13]. Atrial fibrillation is mediated by an increased sensitivity to β1 adrenergic receptors and decreased sensitivity to M2 muscarinic receptors [14], decreased L-type calcium channel activity, increased automaticity in pulmonary vein myocytes with a rise in arrhythmogenicity [15], and increased voltage-gated potassium channel activity with the latter shortening the refractoriness of myocytes, enhancing re-entry circuits [16]. 

Once euthyroidism is achieved, atrial fibrillation reverts to sinus rhythm in 75%-100% of cases, usually after 13-15 weeks of antithyroid therapy [17]. Predictors for persistent atrial fibrillation after the achievement of euthyroidism are advanced age, longer duration of hyperthyroidism, and longer duration of atrial fibrillation. Persistent atrial fibrillation is an indication of cardioversion, and recurrent atrial fibrillation is an indication of ablation. Patients with hyperthyroidism-associated atrial fibrillation respond better to cardioversion with lower recurrence rates compared to patients with non-hyperthyroidism-associated atrial fibrillation [17]. Similarly, recurrence after an arrhythmogenic foci ablation is correlated to high FT3, high FT4, or low TSH levels [12].

Ventricular Fibrillation

Contrary to popular belief, ventricular tachyarrhythmias are more common in patients with hypothyroidism compared to hyperthyroidism [17]. Thus, ventricular fibrillation is a rare association in patients with hyperthyroidism, and the frequency of ventricular fibrillation in hyperthyroidism is equal to that of the general population. Among hyperthyroidism patients, those with Graves’ disease exhibit a higher incidence of ventricular fibrillation compared to toxic nodular goitre [11]. Possible pathophysiologic mechanisms for ventricular fibrillation in hyperthyroidism include coronary vasospasm induced by hyperthyroidism [18] (as well as amiodarone [19]), shortening of repolarisation induced by hyperthyroidism [20], prolonged QTc induced by hyperthyroidism [20] (as well as amiodarone [21]), and hypokalaemia associated with thyrotoxic periodic paralysis [22]. 

Graves’ disease patients with β1-adrenergic and M2 muscarinic autoantibodies are associated with increased incidence of ventricular tachycardia and ventricular fibrillation [12]. Moreover, THs increase the genesis of ventricular arrhythmias through their influence on the autonomic nervous system [12]. As the antithyroid drugs and β-blockers might further shorten the repolarisation period associated with hyperthyroidism to predispose them to developing ventricular fibrillation, hyperthyroid patients with malignant ventricular tachyarrhythmias might require implantable cardioverter defibrillators to prevent sudden cardiac death [20].

Bradyarrhythmias associated with hyperthyroidism 

Bradyarrhythmias, including atrioventricular blocks and sinoatrial blocks, have been rarely described in hyperthyroidism patients [23]. Prolonged intra-atrial conduction and intraventricular conduction, in the form of increased P wave duration and right bundle branch block, are seen in around 15% of hyperthyroid patients [24]. There were only a handful of case reports available in the literature showing an association between CHB and hyperthyroidism. In these case reports, most patients with hyperthyroidism associated with CHB have additional risk factors like infections, rheumatic fever, hypercalcemia, hypokalaemia, or digoxin therapy [25]. Although CHB is usually the result of these primary insults, thyrotoxicosis might aggravate their condition. However, a few patients have hyperthyroidism as the sole aetiology for CHB, and treatment of hyperthyroidism might improve their atrioventricular conduction [25]. 

The pathophysiological mechanisms for hyperthyroidism-associated bradyarrhythmias and CHB are less well understood. It is thought to be mediated by interstitial inflammation of the atrioventricular node, bundle of His, and the bundle branches. It could also be mediated by focal myocarditis around the atrioventricular node. This inflammation could be caused by either a direct effect of THs, an indirect effect of autoimmune response, or mediated by the autonomic nervous system. Hyperthyroidism might be considered a reversible cause of CHB; however, the time lag between the initiation of antithyroid therapy and the recovery of CHB is unclear. Studies have shown that the atrioventricular conduction might improve after six weeks of antithyroid therapy [25]. However, this improvement occurs in only a minority (14%) of patients, compared to a significant improvement in atrial fibrillation after antithyroid therapy [25].

Treatment options for CHB associated with hyperthyroidism are antithyroid therapy, with the addition of corticosteroids in cases of thyroid storm. Rate-controlling drugs like β-blockers, however, are avoided due to the risk of worsening atrioventricular conduction [25]. American College of Cardiology and American Heart Association guidelines for device-based therapy of cardiac rhythm abnormalities recommend permanent pacing for patients with third-degree or advanced second-degree atrioventricular block; the exceptions to this that are mentioned include asymptomatic patients, conduction blocks that are expected to resolve, and unlikely to recur in cases of drug toxicity, Lyme’s disease, ischaemia/infarction, or electrolyte disturbances [26]. However, the guideline does not mention conduction disturbances associated with thyroid dysfunction. 

A position paper from the European Heart Rhythm Association (EHRA) recommended that though restoration of euthyroidism is the primary goal in bradyarrhythmias associated with hyperthyroidism, patients with severe bradyarrhythmias might require the insertion of a temporary pacemaker. However, if the bradyarrhythmias persist despite achieving an euthyroid state, a dual-chamber permanent pacemaker should be inserted [17]. Hence, if the atrioventricular conduction is not improving with antithyroid therapy, patients who were asymptomatic become symptomatic or are developing haemodynamic instability associated with the atrioventricular block, and pacemaker insertion is indicated. There is, however, a lack of consensus regarding the optimal duration of temporary pacing in patients with hyperthyroidism-associated atrioventricular block. Further studies are needed to evaluate the natural history of CHB in patients with hyperthyroidism and the best treatment option [27]. Only 3% of CHBs requiring a pacemaker are truly secondary to hyperthyroidism, implying that this is a rare indication [17].

## Conclusions

This case highlights that although tachyarrhythmias are commonly associated with the hyperthyroid state, bradyarrhythmias can also be encountered, albeit rarely. While the majority of patients with hyperthyroidism and atrial tachyarrhythmias revert to sinus rhythm, only 14% of patients with atrioventricular block improve after achieving a euthyroid state, and the failure of resolution of CHB post achieving a euthyroid state does not always indicate a congenital CHB. Though restoration of euthyroidism is the primary goal in hyperthyroidism with bradyarrhythmia, severe bradyarrhythmia might require temporary pacing, and persisting bradyarrhythmia despite euthyroidism might require permanent pacing.

We appreciate that a congenital CHB cannot be ruled out. However, the absence of maternal autoimmune diseases (lupus, Sjögren's syndrome, and autoimmune thyroid disease) and structural congenital heart disease leans away from the possibility of congenital CHB. Moreover, the fact that the patient was asymptomatic until hyperthyroidism was diagnosed but then became symptomatic, favours the possibility that hyperthyroidism plays a causative role in this patient's CHB.
